# Supplementary material for: High-Throughput Sequencing to Investigate Phytopathogenic Fungal Propagules Caught in Baited Insect Traps
Source: J Fungi (Basel). 2019 Feb 12;5(1):15. doi: 10.3390/jof5010015 (PMC6463110; doi:10.3390/jof5010015)
Supplement: Supplementary file 1 [file jof-05-00015-s001.pdf]

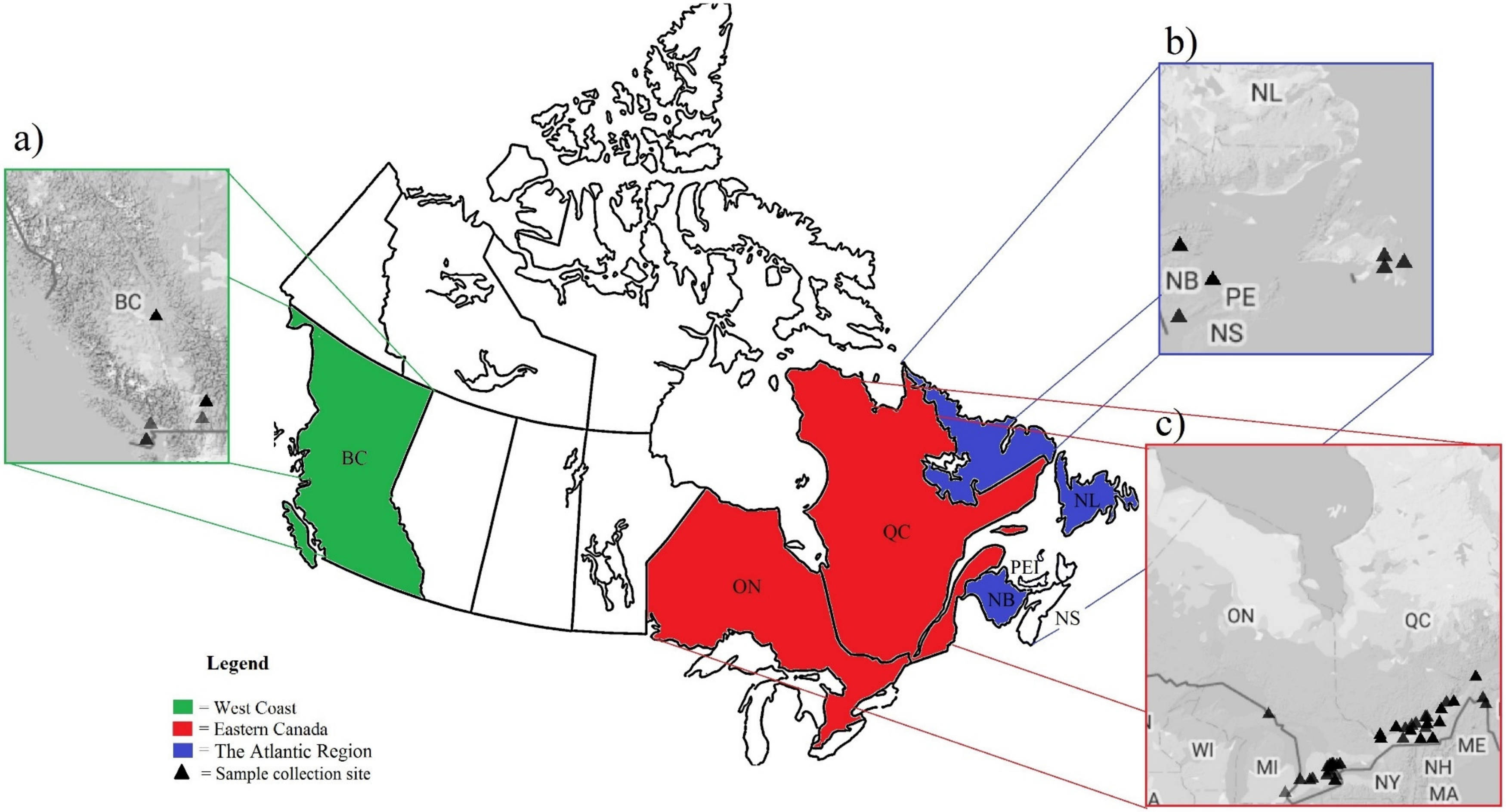

Supplementary Figure S1: Sampling sites in Canada; a) West Coast, b) the Atlantic Region, and c) Eastern Canada. Adapted from Google Earth

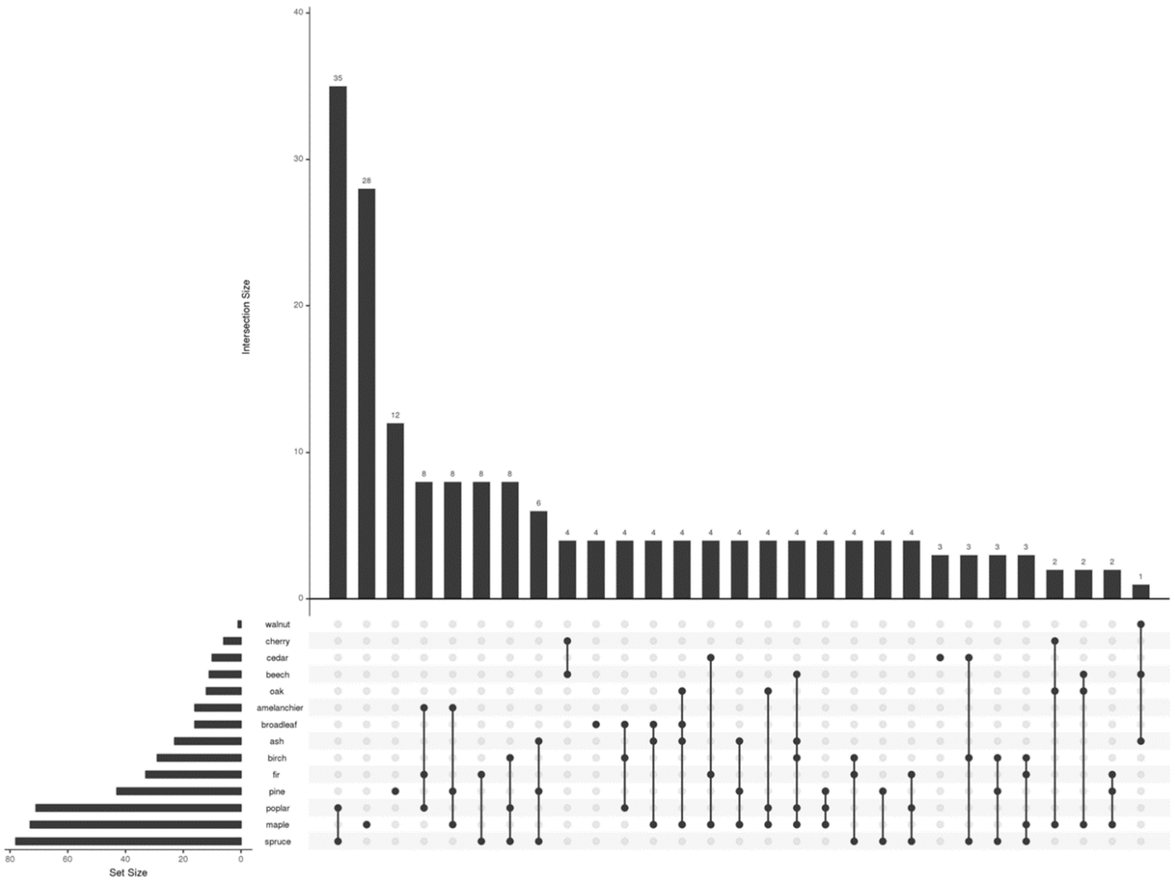

Supplementary Figure S2: Upset plot to visualize a few types of trees that surrounded the sample traps (including the trees from which traps were suspended). The intersection size number represents the number of times a specific tree combination was found (similar to a Venn diagram), and the set size number corresponds to the number of samples surrounded by a specific type of tree. Most samples were collected from traps placed in forested areas comprising more than one tree species.

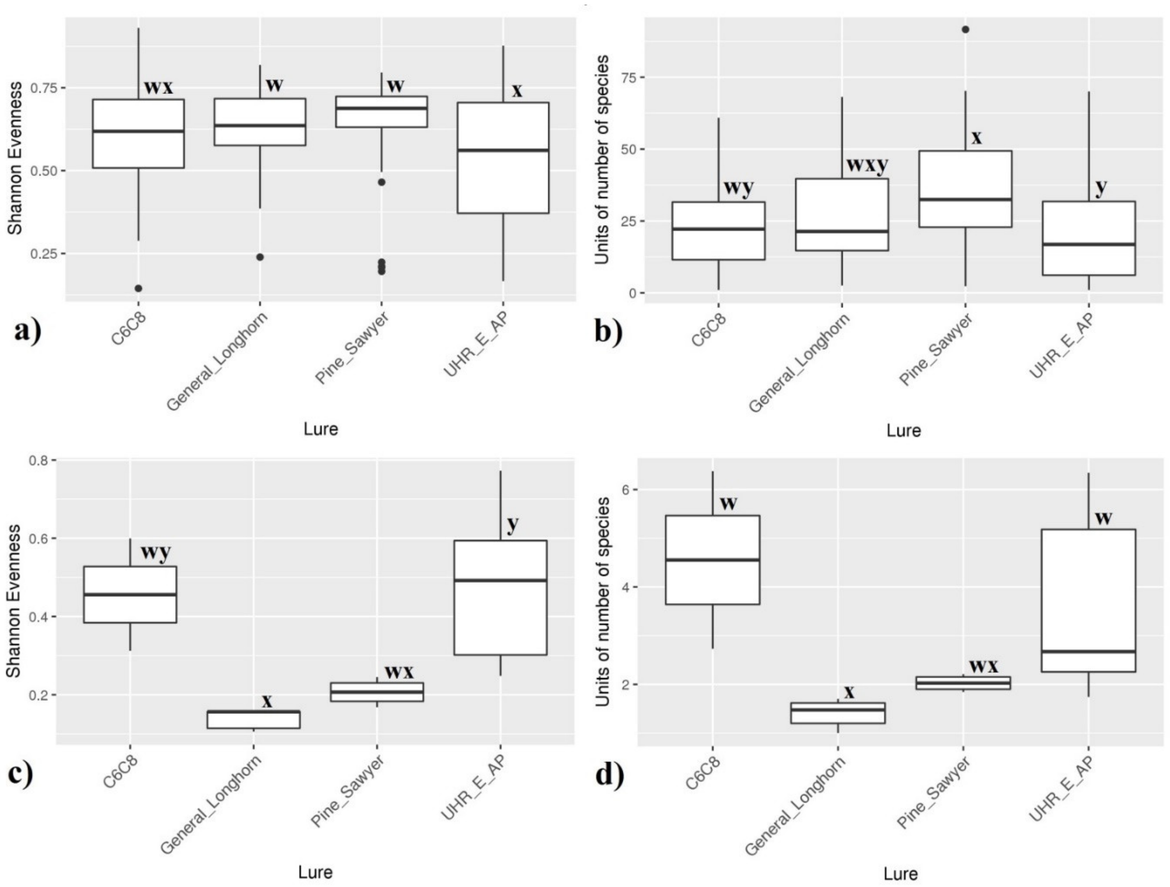

Supplementary Figure S3: Fungal species a) evenness (Shannon) and b) true diversity (Shannon) by semiochemical type, and oomycete species c) evenness (Shannon) and d) true diversity (Shannon) by semiochemical type. The ITS1 sequences were used. Pairwise comparison significance calculated with t tests (P value < 0.05 (α)). Different letters (wxy) show a significant difference between the boxplots compared whereas shared letters represent non-significantly different boxplots.

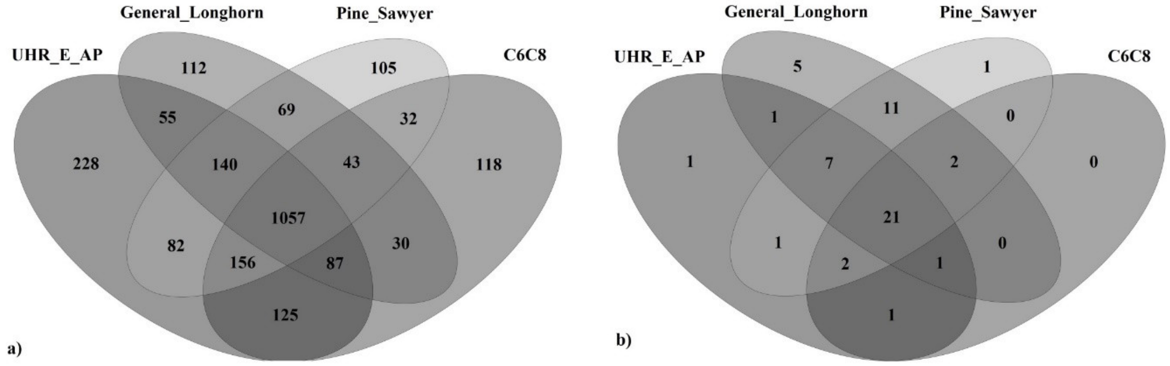

Supplementary Figure S4: Venn diagram of a) fungal species shared or unique to the semiochemical type employed in insect traps prior to species subtraction and b) oomycete species shared or unique to the semiochemical type employed in insect traps prior to species subtraction. All were obtained by amplifying the ITS1 genic region.

Supplementary Material S1: Semiochemicals' composition and additional details.

Each lure set (i.e., C_6C_8, UHR_E_AP, General Longhorn, and Pine Sawyer) was placed on separate traps.

First semiochemical set: C_6C_8.

The first combination (C_6C_8) consisted of racemic 3-hydroxyhexan-2-one (C_6) (Bedoukian Research Inc., Danbury, CT, USA), racemic 3-hydroxyoctan-2-one (C_8) (Bedoukian Research Inc.), and ultra-high release ethanol (UHR EtoH), where each chemical was placed within individual release devices. C_6 and C_8 were verified 99% pure by gas chromatography–mass spectrometry (GC-MS) by the Canadian Forest Service, and loaded into polyethylene pouches by Contech Inc (Delta, BC, Canada). Each pouch contained 1.4 g of either C_6 or C_8. The release rates (at 20°C) were 20 mg/d for C_6, and 25 mg/d for C_8. Two C_6 and two C_8 pouches (semiochemicals) were both placed on a trap to obtain cumulative release rates of 40 to 50 mg/d.

Second semiochemical set: UHR_E_AP.

The second semiochemical set (UHR_E_AP) consisted of UHR EtoH and UHR alpha-pinene. The two chemicals were loaded into separate release devices and placed on an insect trap. The UHR ethanol (95% purity, 121.5 g loaded/pouch) and UHR alpha-pinene [95% (-) enantiomer, 172 g/pouch] lures (Contech Inc., Delta, BC, Canada) had release rates (at 20°C) of 275 mg/d and 2 g/d, respectively. The UHR ethanol and UHR alpha-pinene chemicals were exactly the same throughout this project.

Third semiochemical set: General Longhorn.

The lure set consisted of UHR EtoH, (*E*)-6,10-dimethyl-5,9-undecadien-2-ol (*E*-fuscumol) and (*E*)-6,10-dimethyl-5,9-undecadien-2-yl (*E*-fuscumol acetate). Both *E*-fuscumol and *E*-fuscumol acetate were synthesized by Bedoukian Research Inc. and placed into polyethylene bubble caps by Contech Inc.; 130 mg/bubble cap of *E*-fuscumol (release rate = 1 mg/d), and 200 mg/bubble cap of *E*-fuscumol acetate (release rate = 2 mg/d).

Fourth semiochemical set: Pine Sawyer.

The Pine Sawyer lure set included four separate components consisting of 2-undecyloxy-1-ethanol (monochamol), UHR EtoH, UHR alpha-pinene and racemic 2-methyl-6-methylene-7-octen-4-ol (ipsenol). Monochamol (99.3% purity, 0.025 g/bubble cap) and racemic ipsenol (>99% purity, 0.04 g/bubble cap) were also purchased from Contech Inc. and had release rates (20°C) of 0.2 mg/d and 0.4 mg/d, respectively.

Supplementary Material S2: Fungal OTU, prior to species subtraction.

The most abundant fungi for all semiochemical treatments at the phylum, genus and species (top 10 per semiochemical) taxonomic levels are listed in Supplementary Table S4.

From the 2439 different species OTU detected prior to the species subtraction, 1057 (43%) were common to all semiochemical types, 228 species (9%) were unique to the UHR_E_AP semiochemical, 112 (4.6%) were unique to General Longhorn, 105 (4.3%) were unique to Pine Sawyer, and 118 (4.8%) were unique to the C_6C_8 semiochemical (Supplementary Figure S4a).

To visualize sampling depth, examples of the rarefaction curves obtained for the spore traps (negative control) and their respective original insect dataset, and subtracted insect dataset are shown in Supplementary Figure S5. The spore trap data demonstrated the highest sequencing depth as saturation was obtained for all of the samples tested with a sequence number per species ranging between approximately 5,000 and 25,000 (sequences). The lowest species number obtained by all samples tested was just below 150. In contrast, only part of the samples from both the original insect and the subtracted data reached saturation in their respective rarefaction curves.

Supplementary Material S3: Oomycetes OTU, prior to species subtraction.

The most abundant oomycetes for all semiochemical treatments at the phylum, genus and species (top 10 per semiochemical) taxonomic levels can be found in Supplementary Table S6. Prior to species subtraction, of the total 54 different OTU, there were 21 species detected in all semiochemical types but few were unique to each treatment (Supplementary Figure S4b). *Pythium monospermum* was unique to traps baited with the UHR_E_AP semiochemical. *Pythium oligandrum* was unique to traps baited with the Pine Sawyer semiochemical. Five species were unique to traps baited with the General Longhorn semiochemical (*Peronospora* sp. UPS F-119986, *P. flava*, *P. sparsa*, *Pythium carolinianum*, and *Phytophthora* spp.). No unique species were recovered from traps baited with the C_6C_8 semiochemical. Within the top ten most abundant species identified, all semiochemicals except C_6C_8 (0.67%) had a high percentage of *Peronospora manshurica* (Supplementary Table S6). The UHR_E_AP (26.1%) and C_6C_8 (26.6%) semiochemicals had a considerably higher number of OTU unclassified below genus compared with General Longhorn (traces) and Pine Sawyer (3.02%). Additionally, ATP9-NAD9 OTU from the original dataset generated prior to the species subtraction could only recover *Phytophthora* spp. from traps baited with the C_6C_8 and UHR_E_AP semiochemicals. *Phytophthora cryptogea* could only be detected from traps baited with the UHR_E_AP semiochemical, whereas *P. foliorum*, *Phytophthora* sp. "*kelmania*" [87] and *P. syringae* were associated with both C_6C_8 and UHR_E_AP lures.

Supplementary Table S1: Summary of the samples collected from 2013 to 2015. Specific details associated with samples available upon request.

**Semiochemical**[a]	**Year**	**Number of samples**	**Canadian provinces**
UHR_E_AP	2013	13	British Columbia, New Brunswick, Newfoundland and Labrador, Ontario and Quebec
	2014	26	Ontario and Quebec
C_6C_8	2013	13	British Columbia, New Brunswick, Newfoundland and Labrador, Ontario and Quebec
	2014	23	
General Longhorn	2015	17	
Pine Sawyer		16	
Total:	3	108	5 Canadian provinces

[a]**UHR_E_AP** = Combination of two semiochemicals (ultra-high release (UHR) ethanol and UHR alpha-pinene) that attract a wide range of bark and wood-boring insects [50,56,57].

C_6C_8 = Combination of three semiochemicals (racemic 3-hydroxyhexan-2-one, racemic 3-hydroxyoctan-2-one, and UHR ethanol). The first two chemicals are aggregation pheromones of some longhorned beetles in the Cerambycinae subfamily [55] but, the addition of UHR ethanol increases attraction to other wood-boring insects.

General Longhorn = Combination of three semiochemicals (UHR ethanol, (E)-6,10-dimethyl-5,9-undecadien-2-ol (E-fuscumol), and (E)-6,10-dimethyl-5,9-undecadien-2-yl (E-fuscumol acetate)) used to attract Spondylidinae, Lamiinae, and Scolytinae beetles [58,59,72].

Pine Sawyer = Combination of four semiochemicals (2-undecyloxy-1-ethanol (monochamol), UHR ethanol, UHR alpha-pinene, and racemic 2-methyl-6-methylene-7-octen-4-ol (ipsenol)) used to attract longhorned beetles (*Monochamus*) and bark and ambrosia beetles due to the added ethanol and alpha-pinene [48,60,61].

Supplementary Table S2: Presence or absence of amplification as detected by gel electrophoreses using barcoded bidirectional PCR products from 108 environmental insect samples by targeted organisms, and the percentages of positive reactions obtained respectively with each bidirectional primer used.

Primer used to append barcodes[a]	Organism	Target region[b]	Positive PCR	Total PCR	Positive (%)[c]
ITS1-Forward	Fungi	ITS1	90	108	84
ITS1-Reverse	Fungi		98	108	91
ITS1-Forward	Oomycete		12	108	11
ITS1-Reverse	Oomycete		12	108	11
ATP9-NAD9- forward	*Phytophthora* sp.	ATP9-NAD9	8	20	40
Total	…	…	220	452	Average: 47

[a]Each PCR included a set of primers but, as presented by Tremblay et al. [62], bidirectional sequencing required one primer per direction to append the sample and organism index (i.e., barcode).

[b]ITS1 = internal transcribed spacer 1 and ATP9-NAD9 = adenosine triphosphate synthase subunit 9-nicotinamide adenine dinucleotide dehydrogenase subunit 9 spacer.

[c]Percentage of the number of positive PCR over the total number of reactions done.

Supplementary Table S3: Exotic and native fungal species of interest that are unique to a semiochemical (i.e., post species subtraction), and grouped by the potential damage (= trophic status of concern) associated with those fungi. Also included is a risk level scale in terms of virulence. Identifications based on the ITS1 sequences obtained.

	Known damage	Semiochemical[a]				Presence status[b]	Risk level[c]	Known host(s)	References
		Pine sawyer	General Longhorn	C_6C_8	UHR_E_AP				
**plant pathogen**									
Ambrosiella ferruginea	galleries and wounds caused by insect vector (mycangia)	x				N, C	2	conifers and deciduous trees	[135]
Ciborinia whetzelii	anthracnose and ink spot disease			x		N, C	2	aspen and cottonwood	[136-138]
Colletotrichum fructi	anthracnose				x	N	2	over 30 plant genera	[139,140]
Colletotrichum nymphaeae	anthracnose, leaf spot, and bitter rot				x	N	2	grapevine, pepper, black locust, strawberry, water lily, apple, crab apple and protea	[141-144]
	insect pathogen							citrus orthezia	[145]
Devriesia americana	unknown			x		N	2[d]	unknown	[146,147]
Devriesia strelitziicola	death of leaves				x	E	2	*Strelitzia* spp.	[148-151]
Erysiphe adunca	powdery mildew	x				N, C	2	*Populus* spp. and willow	[94,152]
Erysiphe convolvuli					x	N, C	2	*Calystegia* spp. and *Convolvulus* spp.	[127]

Erysiphe cruciferarum				x		N, C	2	mustard, cabbage, bok choy, and turnip	[153]
Erysiphe diffusa					x	N	2	soybean and legumes	[154,155]
Erysiphe elevata			x			N	2	flowering trees	[156,157]
Golovinomyces depressus			x			E	2	numerous plants in the Asteraceae family	[94,127,152]
Neoerysiphe galeopsidis					x	N, C	2	wild basil, nettle, white turtlehead, and mint	[136,153,158, 159]
Podosphaera clandestina				x		N, C	2	serviceberry, hawthorn, purple loosestrife, crab apple, apricot, cherry, plum, peach and spirea	[102,136,158]
Podosphaera leucotricha					x	N, C	2	apple, and crab apple	[102,136,153]
Podosphaera lini					x	E	2	flax	[127,152,160]
Podosphaera negeri				x		E	2	flowering shrubs	[152,161]
Echinodontium tinctorium	heart rot and brown stringy rot		x			N, C	3	hemlock, fir, and cedar	[102,136,162, 163]
Leptographium sp.	blue stain and sapstain	x				N, C	1?	conifer and hardwood	[104-108,164]
Lirula macrospora	needle cast		x			N, C	3	spruce	[136,165]
Meria laricis		x				N, C	3	larch	[102,166]
Mollisia dextrinospora	eyespot of cereal				x	E	2	cereals	[167-169]
Mycosphaerella areola	areolate mildew		x			N	3	cotton	[127,170]
Phaeo-acremonium inflatipes	wilt and decline	x				N	1?	*Quercus* spp., *Nectandra* spp., whitebeam, vine, and quince	[19,127,171- 173]
Phoma glomerata	blight, leaf spots, and fruit rot			x		N, C	2	over 80 different plants	[127,136,174- 176]

Pucciniastrum circaeae	rust		x			E	2	shrubs, fir and *Circaea* spp.	[127,177]
Septoria gladioli	leaf spot and hard rot				x	N, C	2	flowers and corn	[102,136,178]
Sirococcus conigenus	shoot blight		x			N, C	2	pine, spruce, fir, and hemlock	[102,179,180]
Sirococcus piceicola			x			N, C	2	spruce	[180,181]
Stagonospora pseudopaludosa	leaf spot				x	E	3	grass	[182]
Teratosphaeria xenocryptica					x	E	2	eucalytpus	[183,184]
Phyllosticta minima				x		N, C	3	maple	[94,102,136,185,186]
Strelitziana mali	sooty blotch				x	E	3	apple and vine	[187-189]
Taphrina padi	fruit deformation	x				E	2	cherry	[190-192]
Verticillium isaacii	vascular wilt			x		N	2	artichoke, tomato, spinach, lettuce, cauliflower, eggplant, pepper, and strawberry	[118,193]
**insect gut associated**									
Candida michaelii	N/A[e]			x		N	N/A	handsome fungus beetle	[194]
**loss of wood value**									
Donkioporia albidofusca	white rot	x				U	5	decaying wood	[94,195]
Melastiza chateri			x			U	5	decaying wood	[94]
Perenniporia luteola					x	U	5	decaying wood	[196]
Phlebiopsis sp.		x				N, C	5	decaying wood	[197,198]
Pleurotus ostreatus					x	N, C	5	decaying wood	[102,136,138,158,199]
Pluteus eludens				x		U	5	decaying wood	[94]
Pluteus phlebophorus					x	U	5	decaying wood	[94]
Ramaria pinicola			x			U	5	decaying wood	[94]
Steccherinum oreophilum					x	N, C	5	decaying wood	[136,200]
Trametes cubensis			x			N	5	decaying wood	[94,201-203]
Antrodia albobrunnea	brown rot		x			N, C	5	decaying wood	[204,205]
Sidera lunata					x	E	5	decaying wood	[94,206]

Diatrype disciformis	beech barkspot				x	N, C	3	decaying hardwood trees	[136,158,207]
Hyphodontia microspora	other wood rots		x			E	5	decaying wood	[208,209]
Phellinus ferrugineovelutinus		x				N, C	3	maple and alder	[102,210,211]

[a]**UHR_E_AP** = Ultra-high release ethanol and ultra-high release alpha-pinene.

C_6C_8 = Racemic 3-hydroxyhexan-2-one (K6), racemic 3-hydroxyoctan-2-one (K8), and ultra-high release ethanol.

General Longhorn = Ultra-high release ethanol, (*E*)-6,10-dimethyl-5,9-undecadien-2-ol (*E*-fuscumol), and (*E*)-6,10-dimethyl-5,9-undecadien-2-yl (*E*-fuscumol acetate).

Pine Sawyer = 2-undecyloxy-1-ethanol (monochamol), ultra-high release ethanol, ultra-high release alpha-pinene, and racemic 2-methyl-6-methylene-7-octen-4-ol (ipsenol).

[b] N = the organism is native or reported to be present in North America, C = the organism is native or reported to be present in Canada, E = the organism is not reported or present in North America (exotic), and U = unknown status because information is lacking for Canada and North America.

[c]Risk associated with the organism on a 1 to 5 scale. 5 = a riskless saprophyte fungus, 4 = a saprophyte fungus capable of causing damages to plants 3 = a weakly-virulent pathogenic fungus, 2 = a moderate virulent pathogenic fungus but common in Canada, and 1 = a highly-virulent pathogenic fungus.

[d]Assumption based on the impact of species within the same genus.

[e]Not applicable.

Supplementary Table S4: Operational Taxonomic Units: fungal identification proportion (%) by semiochemical type at the phylum, genus and species (Top 10) taxonomic level using the ITS1 genic region. Data obtained prior to species subtraction.

Semiochemical[a]	UHR_E_AP	C_6C_8	General Longhorn	Pine Sawyer
**Phylum**				
Ascomycota	39.6	41.6	63.9	68.5
Basidiomycota	39.5	40.9	24.2	23.0
Unidentified OTU	17.5	14.2	9.3	5.6
OTU unclassified below kingdom	3.1	3.1	2.4	2.4
Chytridiomycota	0.3	0.1	0.1	0.2
Zygomycota	0.1	0.1	0.1	0.2
Glomeromycota	traces[b]	1.0	traces	traces
Rozellomycota	traces	0.5	absent	traces
**Genus**				
Unidentified OTU	40.5	34.2	29.5	30.0
Rhodotorula	10.0	11.0	traces[b]	3.2
Cystobasidium	7.5	5.5	traces	traces
Cryptococcus	4.3	4.1	5.5	6.1
OTU unclassified below family	3.1	3.1	2.4	2.4
Alternaria	2.9	traces	traces	traces
Epicoccum	2.5	5.2	3.3	2.2
Phoma	2.2	3.2	traces	traces
Scopuloides	1.8	traces	traces	traces
Verticillium	1.8	2.2	traces	traces
Hannaella	traces	2.3	traces	traces
Wickerhamomyces	traces	1.8	traces	traces
Aureobasidium	traces	traces	5.3	traces
Leptographium	traces	traces	5.2	4.4
Cladosporium	traces	traces	3.9	7.3
Neurospora	traces	traces	3.2	traces
Kluyveromyces	absent	traces	3.0	traces
Torulaspora	traces	traces	2.3	traces
Candida	traces	traces	traces	8.0
Mycosphaerella	traces	traces	traces	3.1
Geopyxis	traces	traces	traces	2.0
**Species**				
fungi sp.	17.5	14.2	9.3	5.6
Rhodotorula mucilaginosa	8.2	8.5	traces[b]	2.7
Cystobasidium slooffiae	6.4	3.3	traces	traces

Ascomycota sp.	5.3	4.5	2.8	5.9
OTU unclassified below genus	9.6	3.1	2.4	2.4
Epicoccum nigrum	2.5	5.2	3.3	2.2
Alternaria alternata	2.5	traces	traces	traces
Scopuloides hydnoides	1.8	traces	traces	traces
Verticillium dahliae	1.8	2.2	traces	traces
Cystobasidium pinicola	traces	2.2	traces	traces
Hannaella luteola	traces	1.9	traces	traces
Wickerhamomyces anomalus	traces	1.8	traces	traces
Leptographium piriforme	traces	traces	5.2	4.4
Aureobasidium pullulans	traces	traces	5.1	traces
Cladosporium exasperatum	traces	traces	3.9	6.9
Neurospora terricola	traces	traces	3.2	traces
Kluyveromyces wickerhamii	absent	absent	2.9	traces
Torulaspora delbrueckii	absent	traces	2.3	traces
Candida sp.	traces	traces	traces	7.9
Mycosphaerellaceae sp.	traces	traces	traces	3.0

[a]**UHR_E_AP** = Ultra-high release ethanol and ultra-high release alpha-pinene.

C_6C_8 = Racemic 3-hydroxyhexan-2-one (K6), racemic 3-hydroxyoctan-2-one (K8), and ultra-high release ethanol.

General Longhorn = Ultra-high release ethanol, (*E*)-6,10-dimethyl-5,9-undecadien-2-ol (*E*-fuscumol), and (*E*)-6,10-dimethyl-5,9-undecadien-2-yl (*E*-fuscumol acetate).

Pine Sawyer = 2-undecyloxy-1-ethanol (monochamol), ultra-high release ethanol, ultra-high release alpha-pinene, and racemic 2-methyl-6-methylene-7-octen-4-ol (ipsenol).

[b]Below 0.01% or not in the top 10 for this semiochemical.

Supplementary Table S5: Unique oomycete species detected in the different semiochemicals after proceeding with species subtraction and using the ITS1 genic region.

Semiochemical[a]		
**General Longhorn**	**C_6C_8**	**C_6C_8 and General Longhorn**
Species		
Peronospora farinosa	*Pythium* sp. CAL-2011e	*Pythium* sp. BG01
Peronospora sp. isolate 079405,59		*Pythium* sp. P3862
Peronospora sp. UPS F-119986		No blast hit
Peronospora sparsa		

Peronospora viciae			
Pythium aff. hypogynum			
Pythium sp. AvdB-2012			
Pythium sp. BP2013k			
Pythium sp. CAL-2011f			
Pythium sp. P19300/1/3			
Saprolegnia sp. SAP1			
Total:	11	1	3

[a] **General Longhorn** = Ultra-high release ethanol, (*E*)-6,10-dimethyl-5,9-undecadien-2-ol (*E*-fuscumol), and (*E*)-6,10-dimethyl-5,9-undecadien-2-yl (*E*-fuscumol acetate).

C_6C_8 = Racemic 3-hydroxyhexan-2-one (K6), racemic 3-hydroxyoctan-2-one (K8), and ultra-high release ethanol.

Supplementary Table S6: Operational taxonomic units: oomycete identification proportion (%) by semiochemical type at the phylum, genus and species (top 10) taxonomic levels using the ITS1 genic region.

Semiochemical[a]	UHR_E_AP	C_6C_8	General Longhorn	Pine Sawyer
**Phylum**				
Oomycota	73.9	73.4	99.9	96.9
OTU unclassified below kingdom	26.1	26.6	0.01	3.02
**Genus**				
Peronospora	38.7	8.55	64.1	9.39
OTU unclassified below family	26.1	26.6	0.01	0.30
Phytophthora	18.4	46.5	16.3	absent
Pythium	13.1	14.4	17.5	0.14
Hyaloperonospora	1.90	2.68	0.87	0.15
Plasmopara	1.72	absent	0.13	absent
Basidiophora	0.13	1.34	1.07	0.01
Saprolegnia	absent	absent	traces[b]	traces
**Species**				
Peronospora manshurica	26.7	0.67	61.3	84.9
OTU unclassified below genus	26.1	26.6	traces[b]	3.02
Phytophthora sp.	18.04	46.2	16.3	traces
Peronospora aestivalis	8.20	2.82	1.36	1.47

Pythium sp. CAL-2011e	4.61	11.4	NA[c]	NA
Pythium hypogynum	4.14	1.63	traces	traces
Peronospora alta	2.11	4.59	0.002	3.16
Pythium sp. BG01	1.80	NA	17.1	NA
Plasmopara viticola	1.72	NA	traces	traces
Hyaloperonospora brassicae	1.42	traces	0.75	0.39
Hyaloperonospora parasitica	traces	2.43	0.001	1.10
Basidiophora entospora	traces	1.34	1.07	traces
Pythium catenulatum	NA	0.32	0.23	traces
Peronospora polygoni	0.01	traces	0.49	2.95
Peronospora variabilis	traces	traces	0.40	0.83
Peronospora sepium	traces	traces	0.19	traces
Pythium sp. 3862	traces	traces	traces	0.96
Peronospora arthurii	traces	NA	traces	0.29

[a]**UHR_E_AP** = Ultra-high release ethanol and ultra-high release alpha-pinene.

C_6C_8 = Racemic 3-hydroxyhexan-2-one (K6), racemic 3-hydroxyoctan-2-one (K8), and ultra-high release ethanol.

General Longhorn = Ultra-high release ethanol, (*E*)-6,10-dimethyl-5,9-undecadien-2-ol (*E*-fuscumol), and (*E*)-6,10-dimethyl-5,9-undecadien-2-yl (*E*-fuscumol acetate).

Pine Sawyer = 2-undecyloxy-1-ethanol (monochamol), ultra-high release ethanol, ultra-high release alpha-pinene, and racemic 2-methyl-6-methylene-7-octen-4-ol (ipsenol).

[b]Below 0.01% or not in the top 10 for this semiochemical.

[c]Not applicable or not in the top 10 for this semiochemical.

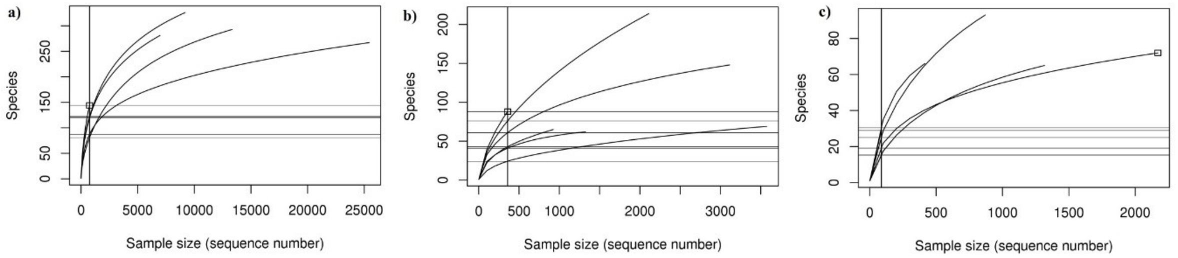

Supplementary Figure S5: Rarefaction curves (number of sequences obtained for each species) for a) spore trap samples and their respective b) insect trap samples (original data), and c) insect trap samples (subtracted data) to visualize sequencing depth.
